# Comparative Analyses of Full-Length Transcriptomes Reveal *Gnetum luofuens*e Stem Developmental Dynamics

**DOI:** 10.3389/fgene.2021.615284

**Published:** 2021-03-25

**Authors:** Chen Hou, Huiming Lian, Yanling Cai, Yingli Wang, Dongcheng Liang, Boxiang He

**Affiliations:** ^1^Guangdong Provincial Key Laboratory of Silviculture, Protection and Utilization, Guangdong Academy of Forestry, Guangzhou, China; ^2^Guangdong Academy of Forestry, Guangzhou, China

**Keywords:** stem development, full-length transcripts, transcription factor, differentially expressed transcripts, gymnosperm

## Abstract

Genus *Gnetum*, of which the majority species are pantropical liana, have broad industrial uses including for string, nets, and paper production. Although numerous studies have investigated anatomical structures during stem development, the underlying molecular mechanisms that regulate this developmental trajectory in *Gnetum* species remain poorly understood. A total of 12 full-length transcriptomes were generated from four stem developmental stages of an arborescent representative of this genus, *Gnetum luofuense*, using Oxford Nanopore Technologies. The results of this analysis reveal a total of 24,151 alternative splicing (AS) and 134,391 alternative polyadenylation events. A remarkably dynamic pattern of AS events, especially in the case of intron retentions, was found across the four developmental stages while no dynamic pattern was found among transcript numbers with varied poly(A) sites. A total of 728 long non-coding RNAs were also detected; the number of *cis*-regulated target genes dramatically increased while no changes were found among *trans*-regulated target genes. In addition, a K-means clustering analysis of all full-length transcripts revealed that primary growth is associated with carbohydrate metabolism and fungi defense, while secondary growth is closely linked with photosynthesis, nitrogen transportation, and leaf ontogenesis. The use of weighted gene co-expression network analysis as well as differentially expressed transcripts reveals that bHLH, GRF, and MYB-related transcription factors are involved in primary growth, while AP2/ERF, MYB, NAC, PLAZ, and bZIP participate in *G. luofuense* stem secondary growth. The results of this study provide further evidence that Nanopore sequencing technology provides a cost-effective method for generating full-length transcriptome data as well as for investigating seed plant organ development.

## Introduction

*Gnetum*, within the order Gnetales, is a gymnosperm genus distributed in tropical and subtropical areas globally (Markgraf, 1930, 1951, Kubitzki, 1990). This genus comprises about 40 species (Price, 1996; Hou et al., 2015); most of these are lianoids with just two, *Gnetum gnemon* L. and *Gnetum costatum* K. Schum, reported to be arborescent (Markgraf, 1951; Hou et al., 2015). *Gnetum* vegetative organs are angiosperm like and species have broad pinnate leaves and swollen nodes at stem connections (Ickert-Bond and Renner, 2015, Hou et al., 2016). Despite the fact that *Gnetum* possesses eudicot-like leaves with paralleled veins, this genus has been characterized by rather low photosynthetic capacity (Feild and Balun, 2008; Deng et al., 2019a). The members of this genus are important economic crops in both Africa and southeast Asia (Markgraf, 1951; Ali et al., 2011, Ingram et al., 2012). Chemical extracts from *Gnetum* leaves and seeds (e.g., stilbenoids and flavonoids) are also of important medicinal value (Ali et al., 2011; Deng et al., 2016, Deng et al., 2017), while stem and bark samples are made into string, nets, and paper (Fu et al., 1999; Isong et al., 1999, Ndam et al., 2001).

**FIGURE 1:**
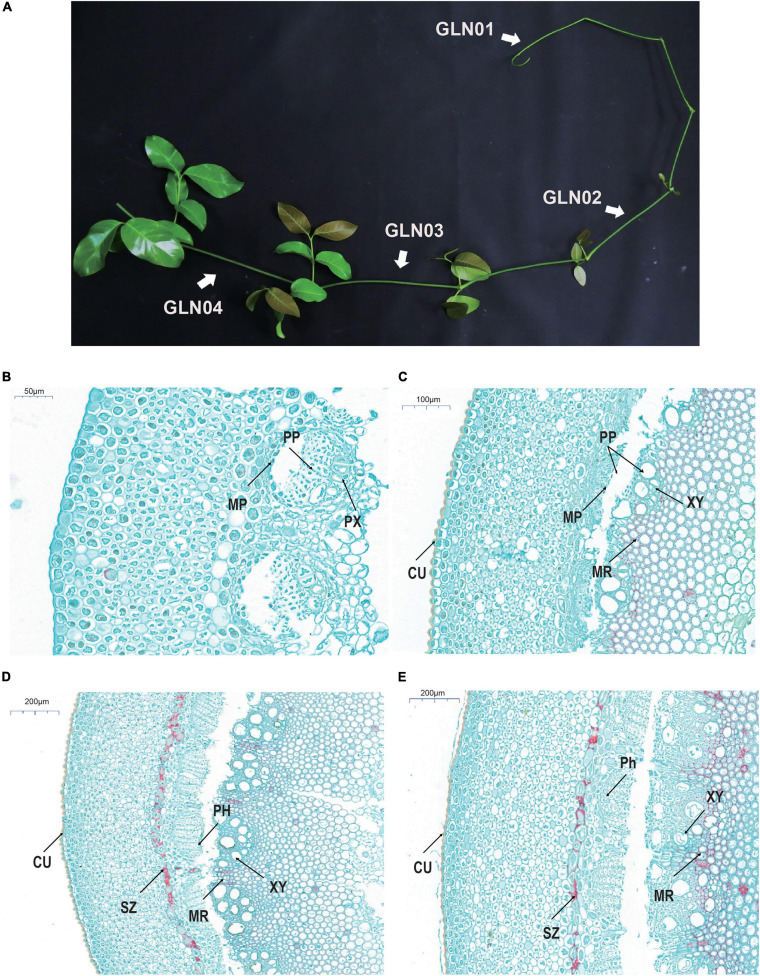
Gross morphology and anatomical structures in *G. luofuense* stems. **(A)** A photograph shows four developmental stages (i.e., GLN01–04) of *G. luofuense* stems identified. **(B–E)** Anatomical structures and histology of *G. luofuense* stems at the developmental stages of GLN01 **(B)**, GLN02 **(C)**, GLN03 **(D)**, and GLN04 **(E)**. Abbreviations: CU, cuticle; MP, metaphloem; MR, medullary ray; PH, phloem; PP, protophloem; PX, protoxylem; SZ, sclerenchymatous zone; XY, xylem.

Stem anatomical structures and development in this genus have been intensively studied encompassing the entire genus and lineages of species (Carlquist and Robinson, 1995, Carlquist, 1996a,b,c, 2012). It is notable that *Gnetum* stem development was documented in a monograph (Maheshwari and Vasil, 1961); this work notes that the transverse sections of young stem samples are more or less rounded (Figure 1A) and that the epidermis in each cases comprises rectangular cells. Four to six cell layers are present beneath this surface that comprises rounded or polygonal units containing chloroplasts. The cortex comprises between 12 and 16 layers comprising thin-walled parenchyma cells. A large number of scattered fibers are then present beneath this surface characterized by a narrow lumen. In slightly older stems, a pronounced sclerenchyma zone rises up from an irregular ring of lignified parenchyma cells within the inner part of the cortex, while collateral and endarch vascular bundles are arranged in a ring shape. These vascular bundles are segregated by medullary rays which comprise mostly tracheids and a few vessels. The phloem comprises sieve and parenchyma cells; in later developmental stages, the cell wall of pith cells becomes lignified and numerous pits emerge.

Although numerous studies have addressed *Gnetum* stem development and anatomical structures, the molecular mechanisms which drive this transition remain poorly understood. Earlier work has emphasized transcription factors (TFs) and has revealed that gene expression of about 70% expressed TFs are upregulated from primary to secondary growth (Dharmawardhana et al., 2010). Another recent study showed that some TFs, e.g., ARF (AUX RESPONSE FACTOR), AP2/ERF, and GRF (GROWTH REGULATION FACTOR), are principally expressed in primary growth, while others, e.g., bZIP (BASIC REGION/LEUCINE ZIPPER), NAC, and PLATS, accumulate during secondary growth in *Populus* (Chao et al., 2019). Among these, AP2/ERF-like TFs have been reported to regulate the expansion and proliferation of *Arabidopsis* cells and shoot architecture (Marsch-Martinez et al., 2006; Mehrnia et al., 2013), while GRFs also determine the formation of shoot meristem and architecture in this genus (Zhang et al., 2018). Similarly, bZIPs are involved in the transition from primary to secondary growth in *Eucalyptus* (Paux et al., 2004) and *Populus* (Dharmawardhana et al., 2010), while the NAC TF has been reported to regulate secondary cell wall development in *Arabidopsis* (Zhong et al., 2006; Hussey et al., 2011). TFs involved in carbohydrate metabolism and plant defense, and protective functions have also been reported during *Populus* primary growth (Dharmawardhana et al., 2010).

In addition to differentially expressed TFs, a growing body of work has also revealed that alternative splicing (AS) and alternative polyadenylation (APA) enrich transcriptome complexity during angiosperm development (e.g., Abdel-Ghany et al., 2016; Liu et al., 2017, Wang et al., 2017; Chao et al., 2018, Chao et al., 2019; Hu et al., 2020) as well as in other gymnosperms (Ye et al., 2019). AS has been proposed as an essential developmental modulator in plants (Wang and Brendel, 2006; Yang et al., 2020). APA promotes the stability, translation, and localization of target RNAs by generating different coding sequences or 3′ UTRs (Elkon et al., 2013). Previous studies on *Gnetum* have shown that various types of AS and APA are involved in *G. luofuense* female strobili and leaf development, but AS and APA events during stem development have never been tested. Previous studies have also shown that *Ginkgo biloba* L. leaves (Wu et al., 2019; Ye et al., 2019) as well as the leaves and female strobili of *G. luofuense* (Hou et al., 2019a) express long non-coding RNAs (lncRNAs) that have *cis*- and *trans*- target genes. LncRNAs are commonly seen in eukaryotic organisms as they are involved in transcriptional and post-transcriptional gene regulation (Mercer et al., 2009; Liu et al., 2015, Karlik et al., 2019). Although full-length transcriptomes have been generated based on merged samples at developmental stages in *Gnetum* (Hou et al., 2019a), dynamic AS and APA event patterns as well as target genes regulated by lncRNAs have been constantly neglected in previous studies.

Oxford Nanopore Technologies (ONT), a new third-generation sequencing technology, is a cost-effective and powerful method. One recent study has shown that this technology generates better quality raw data and estimates transcript levels more accurately than PacBio technology (Cui et al., 2020). ONT has been broadly applied in whole genome sequencing but has rarely been used in full-length transcriptome sequencing. We therefore generated 12 full-length transcriptomes from four developmental stages of *G. luofuense* stems using Nanopore sequencing technology. We first surveyed AS and APA events and identified lncRNAs as well as their dynamic patterns across *G. luofuense* stem development. We then determined deferentially expressed transcripts (DETs) and TFs based on expression levels of each transcript which were then investigated and further validated by qRT-PCR. Our investigation of the molecular mechanisms that regulate vegetative and reproductive organ development provide new insights on *Gnetum* industrial and medical resource exploration.

## Materials and Methods

### Plant Sampling and Anatomic Analysis

Stem material for transcriptomic sequencing was collected from one female and one male individual of *G. luofuense* (voucher nos. CH003 and CH004, respectively, they have been deposited in SYS, Guangzhou, China). Samples were collected from the Bamboo Garden at Sun Yat-sen University, Guangzhou, China, on October 2, 2019. Plant sampling has been permitted by College of Life Science, Sun Yat-sen University. Four developmental stages, GLN01 (stem apex, diameter = 0.5 to 2 mm), GLN02 (2 to 3 mm), GLN03 (3 to 4 mm), and GLN04 (4 to 5 mm), were identified in this study (Figure 1). Three replicate samples from each developmental stage (two from the female individual and one from the male individual) were prepared, resulting in a total of 12 sequencing samples. Identical stem samples were also collected for qRT-PCR analysis.

*Gnetum luofuense* stem samples were dissected into pieces using a knife blade and incubated in formaldehyde–acetic acid–alcohol (FAA) for 96 h. Softened stem material was then subsequently placed into 70% ethanol and further dehydrated in an ethanol series at concentrations of 70%, 96%, and 100%. Subsequent to dehydration, plant material was embedded in paraffin wax and sectioned into thicknesses between 3 and 4 μm using a rotary microtome (Leica, YGQ-3126F, Germany). Sectioned stem material was then extended in water at 40°C and then adhered to a glass slide at 60°C for 15 min. Glass slides were then stained using a safranin O-fast green staining method (Getzy et al., 1982) and were deposited at the Guangdong Academy of Forestry, Guangzhou, China. Observations were then made using light microscopy (Nikon BD-YG3001).

### RNA Extraction and Nanopore Sequencing

All 12 stem samples were incubated in liquid nitrogen and stored at −20°C. Total RNA was then extracted using an RNA kit (Qiagen, Valencia, CA, United States) while relic DNA was removed using RNase-free DNase (Qiagen). We then used 1% agarose gel electrophoresis, a NanoDrop spectrophotometer (Thermo Fisher Scientific, Wilmington, DE, United States), and an Agilent 2100 Bioanalyzer (Agilent Technologies, Palo Alto, CA, United States) to assess the concentration, purity, and integrity of extracted RNA. Furthermore, before full-length transcriptome sequencing, 1 μg total RNA was prepared for cDNA libraries using cDNA-PCR Sequencing Kit (SQK-PCS109) protocol provided by ONT. Libraries were then created using a sequencing library preparation kit. We added defined PCR adapters directly to both ends of the first-strand cDNA. The establishment of cDNA libraries was subject to 14 circles of PCR amplification with LongAmp Tag (NEB). The PCR products were then subjected to ONT adaptor ligation using T4 DNA ligase (NEB). Agencourt XP beads was used for DNA purification according to ONT protocol. The final cDNA libraries were added to FLO-MIN109 flowcells and libraries were then sequenced using a MinION Mk1B sequencer.

### Raw Read Processing and Genome Mapping

Raw data generated from Nanopore sequencing were analyzed using MinKNOW version 2.2 (Oxford, United Kingdom) with a read quality score ≥ 7 and a read length ≥ 500 bp. The Silva rRNA database^1^ was then searched to delete ribosomal RNA from sequenced data. Full-length transcripts (FLs) were identified via the presence of primers at both ends of cleaned reads. The clustering of detected FLs was then detected after mapping to the *G. luofuense* reference genome (genome data have been deposited in https://datadryad.org/stash/dataset/doi:10.5061/dryad.0vm37) using minimap2 (Li, 2018). A consensus isoform was obtained and polished using pinfish^2^. Similarly, consensus FLs were mapped to the *G. luofuense* reference genome using minimap2. To remove redundant FLs, all mapped transcripts were collapsed using the cDNA Cupcake package with a minimum coverage of 85% and a minimum identity of 90%. Consensus FLs with sequence differences at 5′ ends were not considered to be redundant isoforms.

### Functional Annotation and Classification

Non-redundant FLs were annotated using BLASTX v.2.2.26 searches (E-value < 1 × 10^–5^) versus gene ontology (GO^3^), Kyoto Encyclopedia of Genes and Genomes (KEGG^4^), Protein Family (Pfam), Clusters of Orthologous Groups of Proteins (KOG/COG^5^), NCBI non-redundant protein sequence (NR^6^), and Swiss-Prot^7^ databases as well as on the basis of HMMER v.3.1b2 searches (E-value < 1 × 10^–10^) based on Pfam^8^ databases (Finn et al., 2011; Albert et al., 2013). GO enrichment analysis was performed using the GOseq software package implemented in R (R Core Team, 2018) while KEGG enrichment analysis was performed using the KEGG Orthology Based Annotation System (KOBAS, Xie et al., 2011). The annotation of novel isoforms was also completed following these protocols.

### Alternative Splicing and Alternative Polyadenylation Analyses

Subsequent to mapping versus the *G. luofuense* reference genome, AS events were identified using the Astalavista approach (Foissac and Sammeth, 2007). Five types of AS were identified on this basis including an alternative 3′ splice site and an alternative 5′ splice site, as well as exon skipping, intron retention, and mutually exclusive exons. APA events were also analyzed using the TAPIS pipeline (Abdel-Ghany et al., 2016), while FL motifs were detected in the sequence of 50 nucleotides upstream of poly(A) sites using the software MEME (Bailey et al., 2006).

### Identification of Coding Sequences and Long Non-coding RNAs

We identified CDS of polished non-redundant isoforms using the software TransDecoder (Haas et al., 2013) based on log-likelihood score, open reading frame lengths, and comparisons of amino acids versus the Pfam database. Prediction of lncRNAs was performed using four methods, the Coding Potential Calculator (CPC) (Kong et al., 2007), the Coding-Non-Coding Index (CNCI) (Sun et al., 2013), the Coding Potential Assessment Tool (CPAT) (Wang et al., 2013), and Pfam. Subsequent to filtering protein-coding reads, lncRNAs were identified as reads that possessed at least 200 nt and two exons. Identified lncRNAs were then classified as either lincRNA, antisense-lncRNA, sense-lncRNA, or intronic-lncRNA. Target genes that are regulated by identified lncRNAs were predicted using the software LncTar (Li et al., 2015); thus, two types of *cis*- or *trans*- target genes were defined as regulated by lncRNAs in this analysis (Kung et al., 2013; Yang et al., 2014).

### Transcription Factor Identification and Weighted Correlation Network Analysis

We identified TFs and their transcripts which were then assigned to different families using the iTAK software (Zheng et al., 2016). A weighted correlation network analysis (WGCNA) was then conducted using the R package WGCNA version 1.42 (Langfelder and Horvath, 2008). Co-expression networks of TFs across four developmental stages were then constructed with settings defined as CPM values ≥ 1, fold change > 1, minimum module size = 30, and ntop value = 150. A hierarchal clustering tree was also constructed using the Dynamic Tree Cut package in R (Langfelder et al., 2007).

### Differentially Expressed Transcripts and K-Means Clustering Analysis

We mapped FLs onto the *G. luofuense* reference genome and saved matched transcripts with coverage above five. Transcript expression was quantified as counts per million (CPM); thus, CPM = reads mapped to transcripts divided by total reads in one sample × 1,000,000. Differential expression analysis of each pair of developmental stages was then performed using the software DESeq version 1.10.1 (Anders, 2010). Resultant *p* values were then adjusted using the Benjamini and Hochberg approach (Benjamini and Hochberg, 1995) to produce false discovery rates (FDRs). Transcripts with FDR-adjusted *p* values < 0.01 and fold changes ≥ 2 were defined as differentially expressed transcripts (DETs). Out of these DETs, transcript expression of three replicates at each developmental stage was averaged, and denoted GLN01, GLN02, GLN03, and GLN04. Thus, based on the averaged transcript expression, all DETs were subjected to K-means clustering analyses. At the same time, we also performed GO and KEGG enrichment analyses with *q*-values < 0.05.

### Validation of Transcript Expression

We selected nine DETs for transcript expression validation using qRT*-*PCR. Thus, primers were designed using the software Primer Premier 5 (Lalitha, 2000), with information about the qRT-PCR protocol listed in Supplementary Table 1. Two micrograms of RNA was extracted from each *G. luofuense* stem developmental stage. The amplification program used here comprised 2 min of initial denaturation at 95°C, followed by 40 cycles of 20 s at 94°C, 20 s at 58°C, and 20 s at 72°C. The series ended with an extension step at 72°C for 5 min. Gene expression was normalized following the *ACTIN* reference (Hou et al., 2019a). Three replicates were performed for each sample with the mean and SD of qRT-PCR gene expression values calculated in each case. The relative expression of target genes was estimated using the ΔΔCt method (Livak and Schmittgen, 2001) with expression of actin gene as the reference. For each sample of qRT-PCR analysis, we applied three replicates to calculate the mean and SD of gene expression.

## Results

### Anatomic Analysis of *G. luofuense* Stems

Cross-sections of *G. luofusense* stems at each of the four developmental stages considered here were more or less circular. Observations show that at the GLN01 stage, the epidermis is composed of polygonal and rectangular cells (Figure 1B). Indeed, beneath the epidermis, between 12 and 16 layers of thin-walled parenchymatous cells are present, while below them, multiple circular portions also occur that are composed of metapholoems, protophloem, and protoxylem. We know that a pronounced cuticle encloses the stem epidermis at the GLN02 stage (Figure 1C); it is also the case that relatively well-developed protophloem and xylems were seen, separated further by wedge-shaped bundles of medullary rays with lignified cell walls. Anticlinal and periclinal divisions of the metacambium and cambium also significantly enlarged the girth of stems between the GLN02 and GLN04 stages. The phloem was well developed at the GLN03 stage and the numbers of vessels within xylem samples also increased remarkably. A pronounced sclerenchymatous zone derived from an irregular ring of parenchyma cells was also observed in the inner part of the cortex (Figure 1D). We fastened a few cells within the pith and medullary rays with safranin red; this procedure showed that red-stained cells within the last developmental stage, GLN04, became dense and mostly lignified in both medullary stem rays and piths. Four developmental stages were therefore selected to represent the transition from primary to secondary growth on the basis of these anatomic structures.

### Raw Data Processing and Genome Mapping

A total of 48,156,451 clean reads were generated using Nanopore sequencing (Supplementary Figure 1A and Supplementary Table 2); these had a mean length that ranged between 1,209 (GLN023) and 1,345 (GLN012). Among clean reads, a total of 38,635,244 FLs with clear primer sequences at both ends were identified, accounting for between 76.35% (2,541,173; GLN011) and 81.82% (3,839,899; GLN021) of the total, depending on the sample (Supplementary Figure 1B and Supplementary Table 3). Indeed, among FLs, all samples had a high proportion of mapped transcripts, ranging between 99.43% (3,399,666; GLN023) and 99.74% (3,733,604; GLN013) (Supplementary Table 4). A total of 494,859 consensus FLs were determined subsequent to clustering and polishing, ranging between 34,843 (GLN011) and 49,929 (GLN031) (Supplementary Figure 1C and Supplementary Table 5). A total of 261,306 non-redundant FLs were then finally obtained after remapping and deletion of redundant examples; these had mean lengths ranging between 1,398 bp (GLN023) and 1,551 bp (GLN013) (Supplementary Figure 1D and Supplementary Table 6).

### Functional Annotation and the Detection of Novel Transcripts

A total of 60,082 transcripts were annotated functionally in this analysis by searching against the GO, KEGG, COG, KOG, Pfam, NR, and Swiss-Prot databases (Supplementary Figure 2A and Supplementary Table 7). Among annotated transcripts, a total of 59,192 transcripts exhibited significant hits versus the NR database; the largest number of hits occurred in *Picea sitchensis* (16,154 genes; 27.29%), *Amborella trichopoda* (3,588 genes; 6.06%), and *Nelumbo nucifera* (2,046 genes; 3.46%) (Supplementary Figure 2B). GO analysis revealed that 33,808 transcripts could be classified into three categories: “biological processes,” “cellular components,” and “molecular functions” (Supplementary Figure 2C). The most abundant biological process GO terms were classified as “metabolic processes” (15,768 FLs), “cellular processes” (14,694), and “single-organism processes” (9,404), while the most abundant cellular component GO terms were classified as “cell parts” (15,373), “cell” (15,279), and “organelle” (11,006). Similarly, the most abundant molecular function GO terms were classified as “catalytic activity” (11,271), “binding” (13,958), and “transporter activity” (1.730), while the top three annotated FL KEGG terms were classified as “ribosome” (1,118, ko03010), “spliceosome” (961, ko03040), and “carbon metabolism” (881, ko01200). A total of 41,398 novel transcripts were also identified in this analysis; 35,052 (84.67%) were annotated by searching against the seven databases mentioned (Supplementary Figure 2A). The number of annotated transcripts identified using the NR, GO, and KEGG databases are summarized in Supplementary Figures 2B–D.

### AS and APA Analyses

**FIGURE 2:**
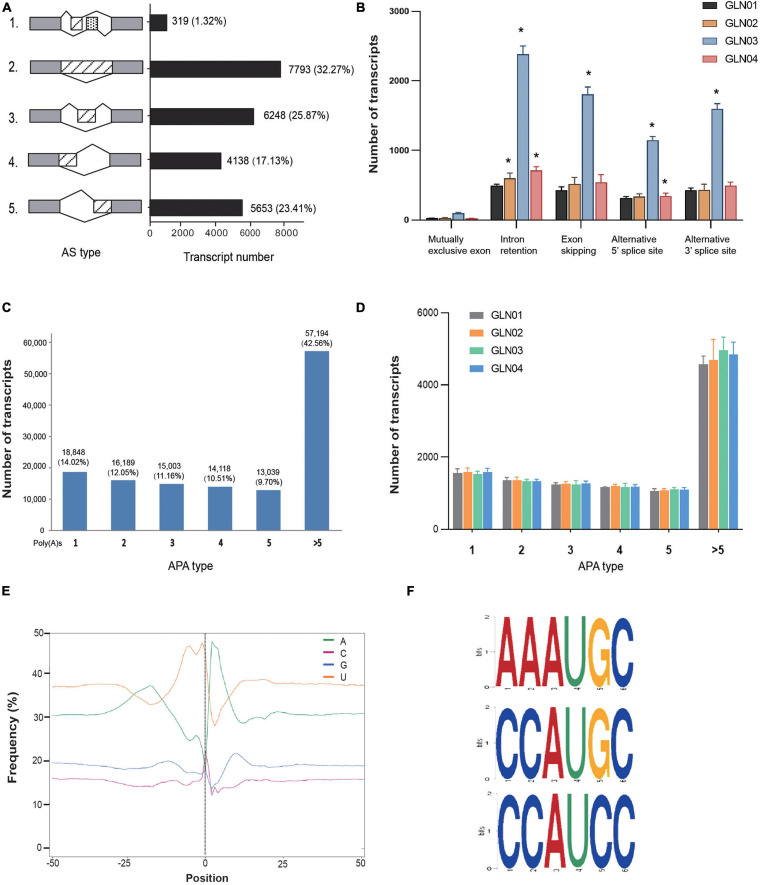
Identification of AS and APA events. **(A)** Numbers of various AS events and their corresponding proportions based on 12 full-length transcripts. **(B)** AS events and corresponding transcript numbers across four developmental stages (GLN01–04) of *G. luofuense* stems. **(C)** Numbers of transcripts that possess various numbers of poly(A) sites based on 12 full-length transcripts. **(D)** APA events and corresponding transcript numbers across four developmental stages (GLN01–04) of *G. luofuense* stems. **(E)** The nucleotide composition around poly(A) cleavage sites, with the relative frequency of each nucleotide on the *y*-axis and the transcriptome composition across all poly(A) cleavage sites on the *x*-axis. **(F)** Three conserved motifs found in the 50 nt upstream region of the poly(A) cleavage site in all full-length transcripts. Symbol * indicates the p values < 0.05 using Student’s t-test analysis.

The results of this analysis showed that a total of 24,151 AS events were detected in the 12 *G. luofuense* stem samples (Figure 2A). Intron retention contributed the largest proportion overall (7.793 events; 33.23%), while a mutually exclusive exon added the smallest proportion (319 events; 1.32%). We then surveyed the numbers of various events throughout the four stem developmental stages (Figure 2B); results showed that intron retention numbers significantly increased between stage GLN02 and stage GLN04 (i.e., Student’s *t*-test with a single tail distribution, *p* values < 0.05). Specifically, alternative 5′ splice site numbers significantly increased between GLN03 and GLN04 while alternative 3′ splice site and exon skipping numbers significantly increased at just GLN03 (*p* values < 0.05). No significant change in events was detected regarding the mutually exclusive exon (*p* values > 0.05).

APA analysis revealed that polyadenylation sites at the 3′ end of FLs scoring more than five encompassed the largest proportion (57,194; 42.56%), followed by those with just one (18,948; 14.02%) or two sites (16,189; 12.05%), respectively (Figure 2C). The numbers of APA events also did not differ significantly throughout *G. luofuense* stem development (i.e., Student’s *t*-test *p* values were all > 0.05) (Figure 2D). Enrichment in uracil (U) was also detected at the 50 nt position upstream of the cleavage site in 3′ UTR while an enrichment in adenine (A) was found at the 50 nt position downstream; this indicates nucleotide bias across all poly(A) cleavage sites (Figure 2E). Three conserved motifs (i.e., AAAUGC, CCAUGC, and CCAUCC) were also detected at the 50 nt position upstream of the poly(A) site in all FLs (Figure 2F).

### CDS and lncRNAs

**FIGURE 3:**
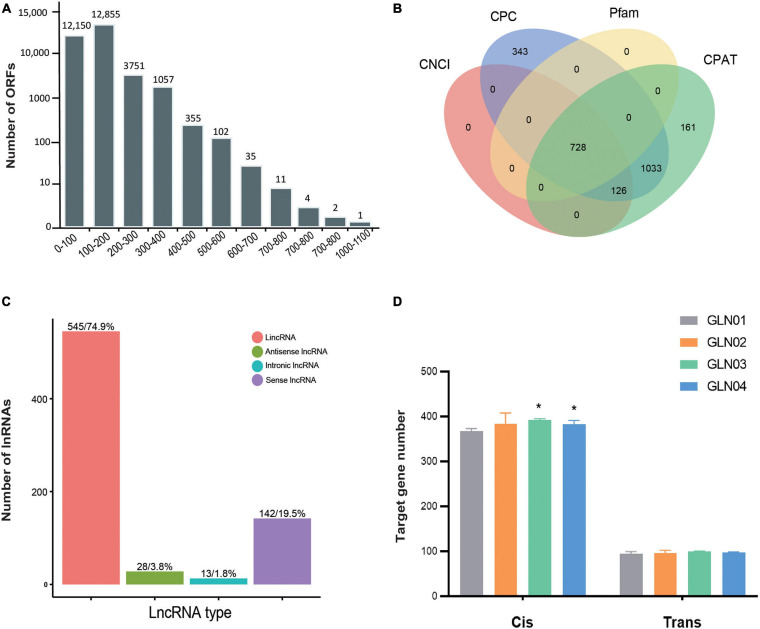
Identification of ORFs and lncRNAs based on 12 full-length transcriptome. **(A)** Length distribution of ORFs detected in all full-length transcripts. **(B)** Venn diagram showing the number of lncRNAs identified using four different approaches: CPC, CNCI, CPAT, and Pfam. **(C)** Functional classification and numbers of four lncRNA types. **(D)** Target genes regulated by lncRNAs in *cis* and in *trans* across four developmental stages. Symbol * indicates the p values < 0.05 using Student’s t-test analysis.

We identified a total of 38,108 ORFs in our samples, including 30,323 (79.57%) that were complete and possessed both start and stop codons. In terms of complete ORFs, lengths between 100 and 200 bp (12,855 ORFs), 0 and 100 bp (12,150), and between 200 and 300 bp (3,751) made up the largest groups (Figure 3A). A total of 728 lncRNAs were also identified with mean lengths ranging between 298 and 4,362 nt; these were determined using four separate methods, CNCI, CPC, Pfam, and CPAT (Figure 3B). These lncRNAs were further classified as comprising 545 lincRNAs (74.86%), 28 antisense lncRNAs (3.85%), 13 intronic lncRNAs (1.79%), and 142 sense lncRNAs (19.50%) (Figure 3C). We also found that the *cis*-target genes regulated by these lncRNAs were considerably larger than those that were *trans-*regulated (Figure 3D). We found significantly increased numbers of target genes *cis*-regulated by these lncRNAs between GLN01 and GLN03 (Student’s *t*-test, *p* = 0.002), and between GLN01 and GLN04 (*p* = 0.037). By contrast, no significant changes (all *p* values > 0.05) were found throughout stem development with regard to *trans*-target gene numbers regulated by these lncRNAs.

### TF and WGCNA Analyses

**FIGURE 4:**
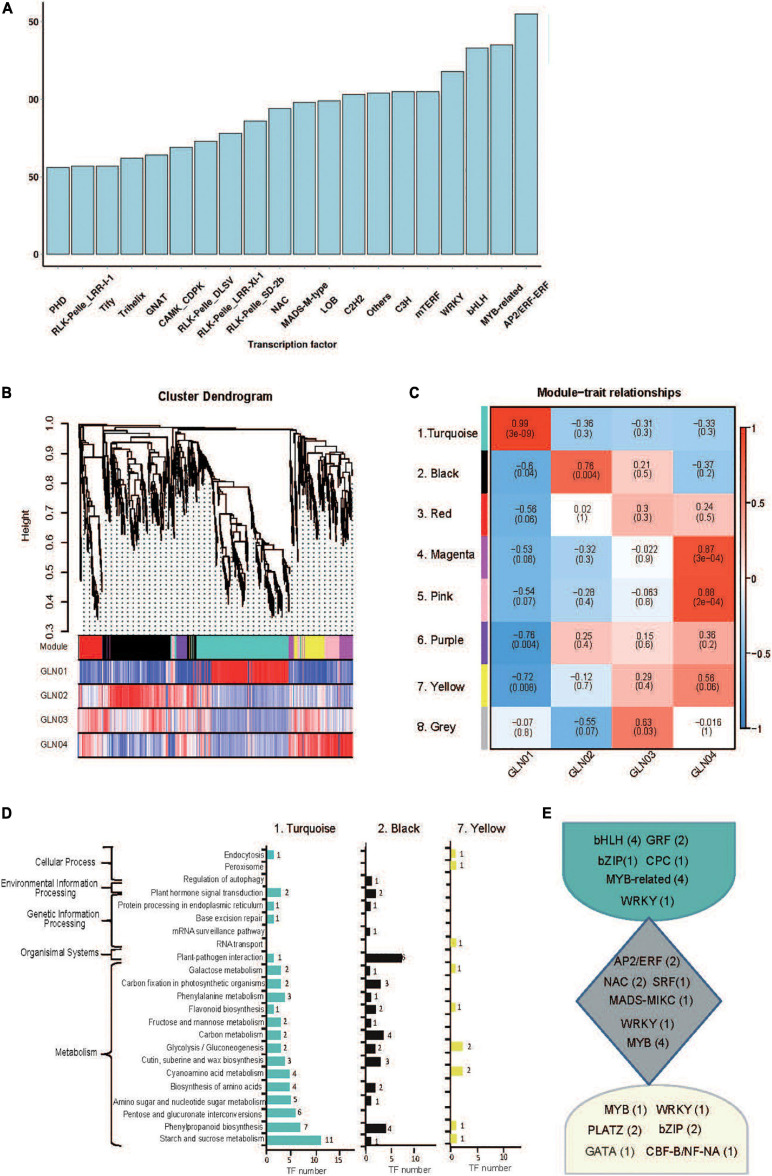
Identification of TFs and results of WGCNA analysis. **(A)** A partial list of TFs (top 20 gene families) identified in 12 full-length transcriptome of *G. luofuense* stems. **(B)** Hierarchical clustering tree showing co-expression modules based on WGCNA analysis. Each branch in the phylogenetic tree corresponds to an individual TF, and highly interconnected TFs are grouped into eight modules. The different colored rows below the phylogeny indicate differentially expressed TFs in *G. luofuense*. **(C)** A heatmap of TF expression patterns in a matrix shows module–trait relationships. The expression patterns of eight modules are shown by the heatmap with a color bar of expression levels from high (red) to blue (red). The correlation coefficient was present for each cell, with the subtended by values of –log(*P*), where *P* is the Fisher’s exact test *P* value. **(D)** KEGG pathway annotations of TFs in the largest three modules. **(E)** Highly expressed TFs and numbers in brackets in the largest three modules.

A total of 4,251 TFs belonging to 208 gene families were identified on the basis of non-redundant FLs. The most abundant of these were AP2/ERF (176), MYB-related (135), and bHLH (133) (Figure 4A), while eight modules of highly correlated TFs through *G. luofuense* stem development were also identified using the WGCNA software (Figures 4B,C). Turquoise (No. 1, 315 TFs), black (No. 2, 215 TFs), and yellow (No. 7, 75 TFs) were the most abundant of these TF modules; TFs in the turquoise module were largely expressed in GLN01 while those in black and yellow modules were densely expressed in GLN02 and GLN04, respectively (Figures 4B,C). It is clear that TFs in the turquoise module were mainly enriched in three KEGG pathways, “starch and sucrose metabolism” (11 TFs, ko00500), “starch and sucrose metabolism” (eight TFs, ko00500), and “pentose and glucoronate interconversions” (six TFs, ko00040) (Figure 4D). Similarly, TFs in the black module were primarily enriched in “plant–pathogen interactions” (six TFs, ko04626), “carbon metabolism” (four), and “phenylpropanoid biosynthesis” (four TFs, ko00940), while TFs in the yellow module were mainly enriched in “cyanoamino acid metabolism” (two, ko00460) and “glycolysis/gluconeogenesis” (two TFs, ko00010). Moreover, we found, e.g., bHLH, GRF, and MYB-related were highly expressed in the turquoise module; e.g., AP2/ERF, NAC, and MYB were in the black module; and MYB, bZIP, and PLATZ were in the yellow module (Figure 4E).

### DETs and K-Means Clustering

**FIGURE 5:**
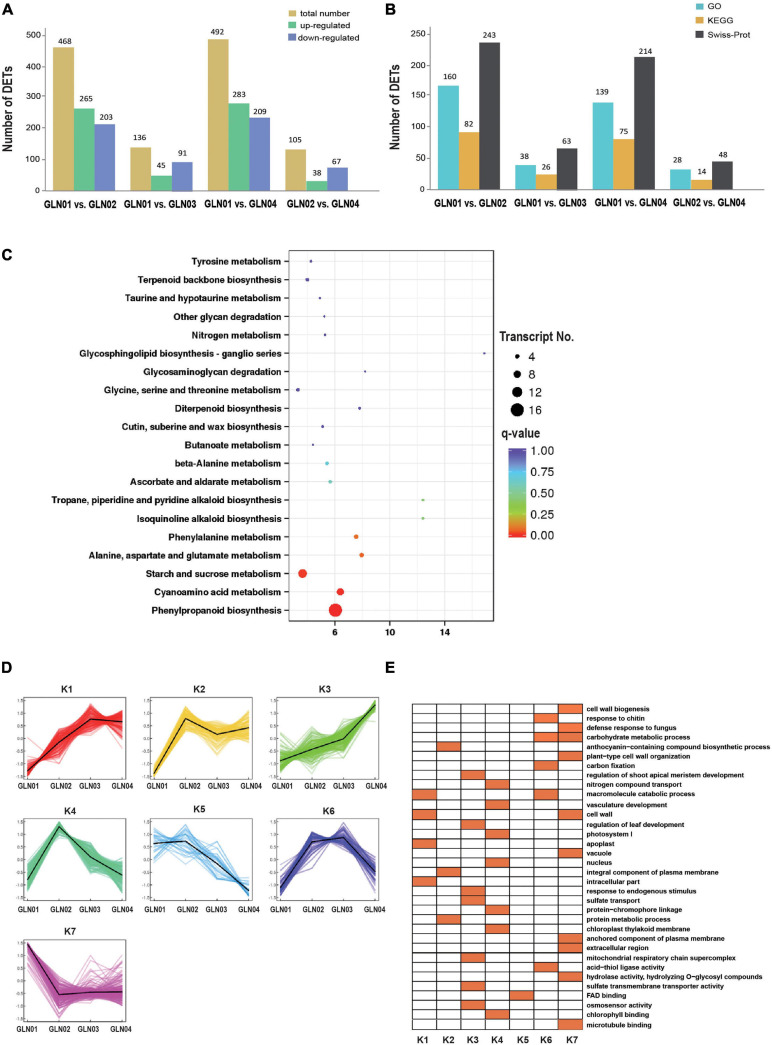
Detected DETs and K-mean clustering analysis of DETs. **(A)** Numbers of DETs between different developmental stages of *G. luofuense* stems. **(B)** Numbers of DETs annotated by the three databases, i.e., GO and KEGG pathway annotations of TFs in the largest three modules, and Swiss-Prot. **(C)** An air bubble graph showing KEGG pathways enriched in the DEGs between GLN01 and GLN04. Gene numbers (circle size) and enrichment *q*-values (circle color) are shown. Warm colors represent *q*-values < 0.05. **(D)** Seven clusters identified based on expression levels of DETs from GLN01 to GLN04. **(E)** GO enrichment among the seven clusters with orange boxes denoting significant enrichment.

Results showed that there were 492 DETs between GLN01 and GLN04 including 283 that were upregulated and 209 that were downregulated (Figure 5A). The second and third largest DET numbers then comprised 468 between GLN01 and GLN02 and 136 between GLN01 and GLN03. The numbers of DETs subsequently annotated by searching against GO, KEGG, and Swiss-Prot databases are presented in Figure 5B. Results revealed that the DET set between GLN01 and GLN04 was significantly enriched in the three KEGG pathways (i.e., *q*-value < 0.05) “phenylpropanoid biosynthesis” (16 DETs), “starch and sucrose metabolism” (10 DETs), and “cyanoamino acid metabolism” (eight DETs) (Figure 5C).

All the DETs identified in this analysis were then grouped into seven clusters using the K-means clustering algorithm (Figure 5D). Results show that transcripts from the K7 cluster were predominantly expressed at the apex (GLN01) while the remaining clusters, K3 and K4, were predominantly expressed between GLN02 and GLN04. Transcripts of these seven clusters were enriched in multiple GO terms (Figure 5E); in one example, K7 cluster transcripts were enriched in “cell wall biogenesis,” “plant-type cell wall organization,” and “cell wall” terms. In another example, transcripts from the K3 cluster were enriched in “regulation of shoot apical meristem development,” “regulation of leaf development,” and “response to endogenous stimulus” terms while transcripts from the K4 cluster were enriched in “nitrogen compound transport,” “vasculature development,” and “photosystem I” terms.

### qRT-PCR Analysis

**FIGURE 6:**
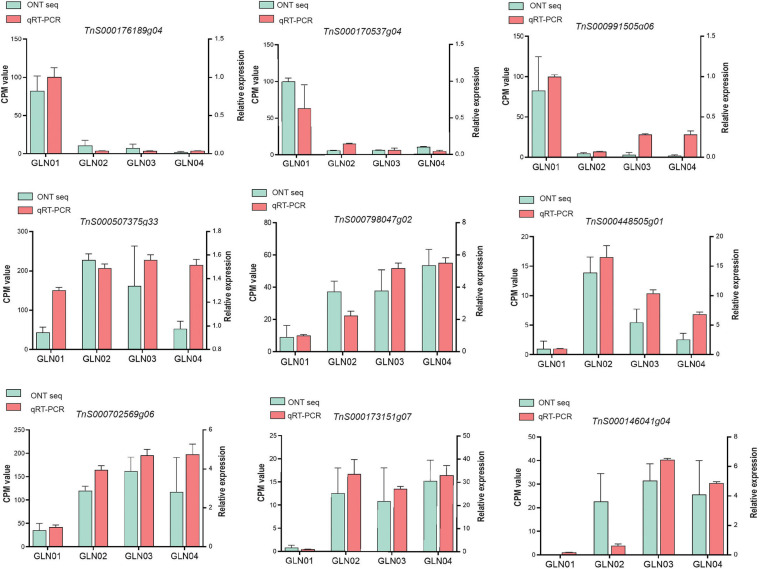
qRT-PCR was used to verify the expression patterns of nine selected genes, using *ACTIN* as an internal control. Counts per million (CPM) values from Nanopore sequencing are indicated on the left *y*-axis and relative qRT-PCR expression levels are indicated on the right *y*-axis.

We performed qRT-PCR experiments to validate the nine genes of interest in the results of DET and K-means clustering analyses (Figure 6). Our results showed that genes *TnS000170537g04*, *TnS000176189g04*, and *TnS000991505g06* were predominantly expressed during the GLN01 stage while the remaining six were predominantly expressed between GLN02 and GLN04.

## Discussion

### Stem Development Transcriptomic Dynamics

In terms of AS event analysis, results show that intron retention accounts for the largest proportion (32.23%) of the total number of events recorded in *G. luofuense* stem FLs (Figure 2A). This number turns out to be smaller than detected in the female strobili (46%) and the leaves (41.5%), respectively (Hou et al., 2019a), but larger than that in the seeds (29.9%) of *G. luofuense* (Deng et al., 2020). These AS mode proportions have also been reported in *Ginkgo biloba* L. (Ye et al., 2019) vegetative and reproductive organs as well as in maize and sorghum (Wang et al., 2018). Intron retention numbers also significantly increased throughout *G. luofuense* stem development (Figure 2B). It is noteworthy that the results reported here differ from those of a previous study in which the percentages of intron retention at the pre-fertilization stage decreased when compared with the post-fertilization stage (Li et al., 2017). We also found that exon skipping numbers as well as alternative 5′ splice and alternative 3′ splice sites at GLN03 all significantly increased compared with the GLN01 stage. These results suggest that AS events are likely to be essential in regulating *G. luofuense* stem development.

The results of APA analysis differ from those of previous studies where transcript numbers declined from one poly(A) site to five poly(A) sites (Figure 2C). At the same time, transcript numbers with more than five poly(A) sites make up a relatively small proportion in *G. luofuense* FLs within the leaves (3.12%), the female strobili (11.14%), and the seeds (3.62%) of *G. luofuense* (Deng et al., 2019b; Hou et al., 2019a, Deng et al., 2020). We further examined transcript numbers and found them consistent regardless of APA mode (Figure 2D) and were also able to show that the patterns of these events are more conserved compared with their AS counterparts. Three conserved motifs (i.e., AAAUGC, CCAUGC, and CCAUCC) were also identified in the 12 *G. luofuense* stem FLs considered in this analysis. Results also differ with regard to the three motifs (i.e., AAAACA, GGGCGC, and CGCCGC) detected in the female strobili of *G. luofuense* (Hou et al., 2019a). Our results suggest that the vegetative and reproductive organs of *G. luofuense* experience variable APA mechanisms during organ development.

Our results show that the short ORFs (length < 200 bp) constituted the largest proportion (82.46%) of the detected ORFs (Figure 3A). It might be because short ORFs lead to high substitution rates as an adaptation to tropical habitats. A similar scenario has been reported among the chloroplast genes in Gnetales (Wang et al., 2015). In addition, it is noteworthy that the proportion of lincRNA in stems (74.9%) differs from the proportion seen in either leaves (33.49%) (Hou et al., 2019a) or female strobili (40.80%) (Hou et al., 2019a) in *G. luofuense* as well as in the leaves of *G. biloba* (50.6%) (Ye et al., 2019). Besides, because our results show the different numbers of *cis*- and *trans*-regulated genes across the entire procedure of stem development in *G. luofuense* (Figure 3D), we therefore suggest that *cis*-regulation of target genes by lncRNA is more important than *trans-*regulation across *G. luofuense* stem development. It is interesting that 44 lincRNAs in *Gossypium hirsutum* were shown to be differentially expressed under salt stress in a recent study; these lincRNAs are mostly *cis*-regulated (Deng et al., 2018). Additional work will be required to investigate the functions of these lincRNAs as well as the mechanisms by which they regulate genes in *G. luofuense* stem development.

### Genes and TFs Related to Primary Growth

The results of this analysis also show that the DETs are enriched in starch and sucrose metabolism (Figure 5C), while those belonging to cluster K7 are enriched in “carbohydrate metabolic processes” GO terms (Figure 5D). TFs in the turquoise module were also annotated with “starch and sucrose metabolism” and “glycolysis/gluconeogenesis” (Figure 4D). These results indicate that the emergence of an apical apex in *G. luofuense* stems requires relatively abundant carbohydrates as a resource for further growth. Our results corroborate those of a previous study that showed the primary growth of *Populus* stems requires carbohydrate metabolism and plant defense (Dharmawardhana et al., 2010). Indeed, another previous study also reported that the regulation of carbohydrate metabolism facilitates cell division and expansion to promote shoot ontogenesis (Morris and Arthur, 1985; Fleming, 2006, Eveland and Jackson, 2012).

The results reported here also show that DETs can be annotated with the KEGG pathway “cyanoamino acid metabolism” (Figure 5C). Similarly, DETs in the K7 cluster were annotated with the GO term “defense response to fungus” (Figure 5D). These differences might be because apical stem tissues lack a physical barrier between them and so the cuticle is potentially vulnerable to external pathogen and fungi. This scenario is similar to the vulnerability seen in fertile and sterile *G. luofuense* and *G. gnemon* reproductive units during pollination periods (Hou et al., 2019b). In terms of detected TFs, the gene *TnS000176189g04* encodes a MYB-related TF and was shown to be principally expressed at GLN01 using qRT-PCR analysis (Figure 6). Another recent study also reported that the *MYB* gene is associated with fungal affection and host cell death induced by jasmonic acid (Lee et al., 2001). This means that MYB-related TFs might trigger a fungi or pathogen-related defense mechanism that aids in *G. luofuense* apical stem development.

Our results reveal that K7 DETs were enriched with the GO terms “cell-wall biogenesis,” “plant-type cell-wall organization,” “vacuole,” and “cell wall” (Figure 5E). In addition, TFs were also enriched in “cutin, suberin, and wax biosynthesis” (Figure 4B). These results are congruent with the emergence of a cuticle on the stem epidermis as well as with the transition from protophloem and protoxylem to phloem and xylem during stem development (Figure 1). In terms of detected TFs, one *bHLH* gene (*TnS000170537g04*) and one *GRF* gene (*TnS000991505g06*) were principally expressed at the GLN01 stage and dramatically declined between GLN02 and GLN04 (Figure 6). The results of this analysis further corroborate a previous study which noted that bHLH TFs are involved in early xylem development in *Arabidopsis* roots under the various auxin conditions (Ohashi-Ito et al., 2013). A further example noted here also showed that cell division in root apical meristems is triggered by a bHLH complex via cytokinin action (Ohashi-Ito et al., 2014). It has also been reported that GRF-interacting factor1 determines the emergence of shoot meristem as well as maize architecture (Zhang et al., 2018). Thus, TF bHLH and GRFs are most likely to regulate *G. luofuense* stem apical shoot development.

### Genes and TFs Related to Secondary Growth

The results of this analysis show that DETs are mainly enriched in the GO terms “regulation of shoot apical meristems,” “response to endogenous stimuli,” and “regulation of leaf development” in the K3 cluster (Figure 5D). Indeed, the DETs were principally expressed at the GLN02 stage and can be annotated with GO terms related to photosynthesis and nitrogen transportation. Our results suggest that stem growth in *G. luofuense* is accompanied by the strengthening of photosynthetic capability and nutrition transportation. The statement is congruent with a general conclusion that woody tissue photosynthesis facilitates bud and trunk development in young plants (Saveyn et al., 2010). It is noteworthy that *G. luofuense* leaves emerge from the stem stage during GLN02 and gradually develop between GLN03 and GLN04 (Figure 1A); *G. luofuense* therefore also probably resorts to the photosynthetic products provided by laterally branched leaves during stem development.

A number of TFs and genes might be involved in *G. luofuense* stem the secondary growth. The gene *TnS000507375g33* that encodes AP2/ERF was upregulated and expressed between GLN02 and GLN03 compared with GLN01 (Figure 6); this implies an important role in secondary growth. It has also been reported that a *AP2/ERF*-like TF participates the expansion and proliferation of *Arabidopsis* leaves (Marsch-Martinez et al., 2006), while additional previous work shows that another *AP2/ERF*-like TF is involved in cell proliferation and influences shoot architecture in *Arabidopsis* (Mehrnia et al., 2013). The gene *TnS000507375g33* might therefore trigger cell proliferation and anticlinal/periclinal expansion in *G. luofuense* stems between GLN02 and GLN04; the *PLAZ* gene *TnS000798047g02* and the *NAC* gene *TnS000448505g01* have been shown to be differentially expressed between GLN01 and GLN04 (Figure 6). It has also been reported that PLAZ and NAC TFs are likely to be important in *Populus* stem secondary growth (Chao et al., 2019), while other studies have shown that a NAC TF is able to negatively regulate *Arabidopsis* xylem fiber (Ko et al., 2007) and secondary cell wall development (Zhong et al., 2006; Hussey et al., 2011). We therefore assume that NAC TFs might facilitate the lignification of xylem fibers as well as the emergence of a sclerenchymatous zone between GLN03 and GLN04.

The results of this analysis reveal that the expression of one *bZIP* gene *TnS000702569g06* was significantly upregulated between GLN02 and GLN04 (Figure 6). This is consistent with the results of a previous study which reported that the morphology of tobacco plants is regulated by a ZIP transcriptional activator via the proportion of the endogenous hormone gibberellin (Fukazawa et al., 2000). Additional studies have also shown that bZIP plays an important role in the transition from primary to secondary growth in *Populus* (Dharmawardhana et al., 2010) and *Eucalyptus* (Paux et al., 2004). Expression levels of the two MYB genes *TnS000173151g07* and *TnS000146041g04* were also both dramatically upregulated between GLN02 and GLN04 (Figure 6), and another earlier study also showed that a hybrid aspen used differentially expressed MYB TFs to regulate the vasculature development of secondary stem growth (Karpinska et al., 2004). In a further example, one MYB TF PtrMYB152 has been shown to participate in the biosynthesis of secondary cell walls in *Arabidopsis* (Wang et al., 2014), while a further MYB TF GhMYB25 manipulates the development of fiber and trichome in this genus (Machado et al., 2009). We therefore assume that *bZIP* and *MYB* genes might be involved in the transition from primary to secondary growth by promoting xylem development as well as the emergence of a sclerechymatous zone.

## Conclusion

We generated 12 full-length transcriptome of *G. luofuense* stems at four developmental stages using Nanopore sequencing technology. The 12 full-length transcriptome of *G. luofuense* stems predicts a total of 24,151 AS events and 134,391 APA events and 728 lncRNAs. WGCNA and K-means clustering analyses revealed that key transcription factors were associated with a series of KEGG pathways including photosynthesis, nitrogen transportation, and leaf ontogenesis. Transcription factors, e.g., bHLH, GRF, and MYB-related transcription factors, participate in primary growth, while others, e.g., AP2/ERF, MYB, NAC, PLAZ, and bZIP, are involved in *G. luofuense* stem secondary growth. These findings provide a valuable the information to the *Gnetum*-related fiber and paper industry, and shed light on the utility of Nanopore sequencing technology for the investigation of full-length transcriptomes in gymnosperms.

## Data Availability Statement

The datasets presented in this study can be found in online repositories. The names of the repository/repositories and accession number(s) can be found below: https://www.ncbi.nlm.nih.gov/bioproject/PRJNA647987/.

## Author Contributions

CH and BH conceived and designed the experiments. CH, HL, YC, and YW performed the experiments. CH, DL, and YC analyzed the data. CH wrote the manuscript. All authors have read and approved the manuscript.

## Conflict of Interest

The authors declare that the research was conducted in the absence of any commercial or financial relationships that could be construed as a potential conflict of interest.

## Footnotes

1 https://www.arb-silva.de

2 https://github.com/nanoporetech/pinfish

3 http://www.geneontology.org

4 http://www.genome.jp/kegg/

5 http://www.ncbi.nlm.nih.gov/COG/

6 http://www.ncbi.nlm.nih.gov/

7 http://www.expasy.org/sprot/

8 http://pfam.xfam.org/

## Supplementary Material

Supplementary Figure 1 Quality control and information of full-length reads using GLN011 as an example. **(A)** Length distribution of clean reads. **(B)** Length distribution of full-length (FL) reads. **(C)** Length distribution of consensus reads. **(D)** Length distribution of non-redundant FL reads.

Supplementary Figure 2 All gene and novel gene annotation on the basis of seven databases. **(A)** Gene number annotated by the seven databases. **(B)** NR homologous species distribution diagram of all genes and novel genes. **(C)** Distribution of GO terms for all annotated genes and novel genes. **(D)** KEGG pathways of all genes and novel genes annotated in the full-length transcriptome of *G. luofuense*.

Supplementary Table 1 Primer information and qRT-PCR systems.

Supplementary Table 2 Clean reads generated by Nanopore sequencing.

Supplementary Table 3 Numbers and percentages of FL reads.

Supplementary Table 4 Mapped FL reads.

Supplementary Table 5 Information of consensus reads.

Supplementary Table 6 Statistics of non-redundant consensus transcripts.

Supplementary Table 7 Annotation and classification of detected ORFs by the seven datasets.
