# Supplementary figures and images for: Comparative Analyses of Full-Length Transcriptomes Reveal Gnetum luofuense Stem Developmental Dynamics
Source: Front Genet. 2021 Mar 25;12:615284. doi: 10.3389/fgene.2021.615284 (PMC8027257; doi:10.3389/fgene.2021.615284)

**A**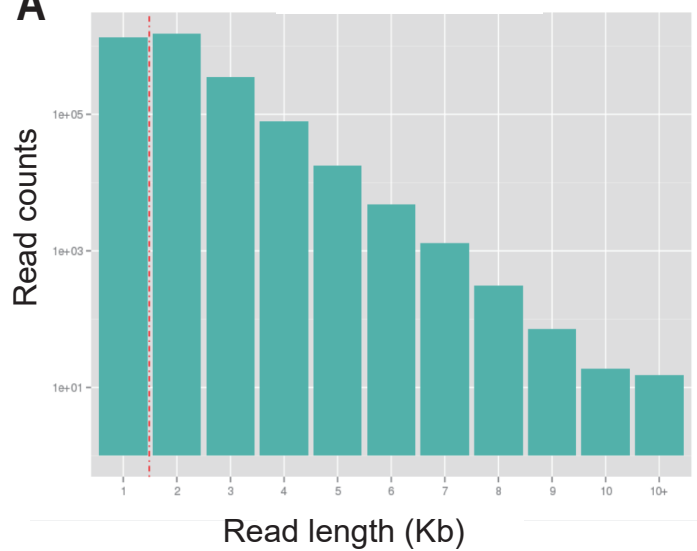**B**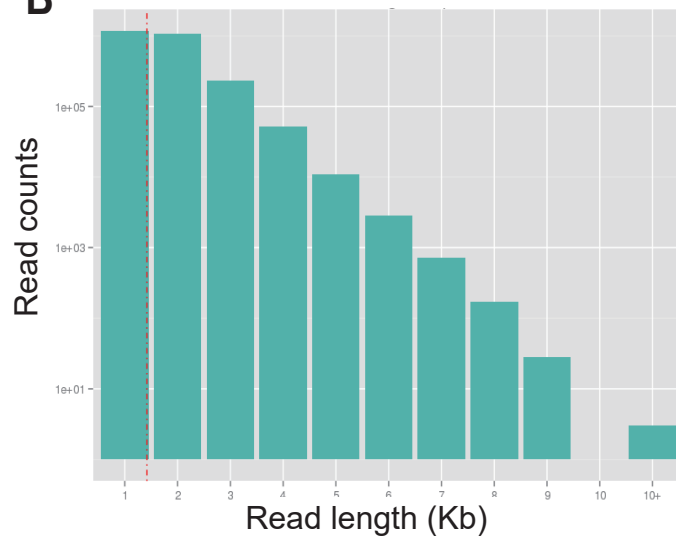**C**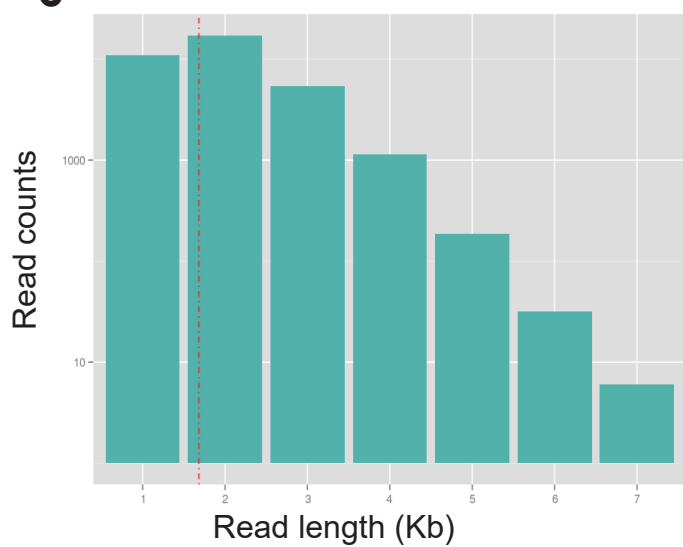**D**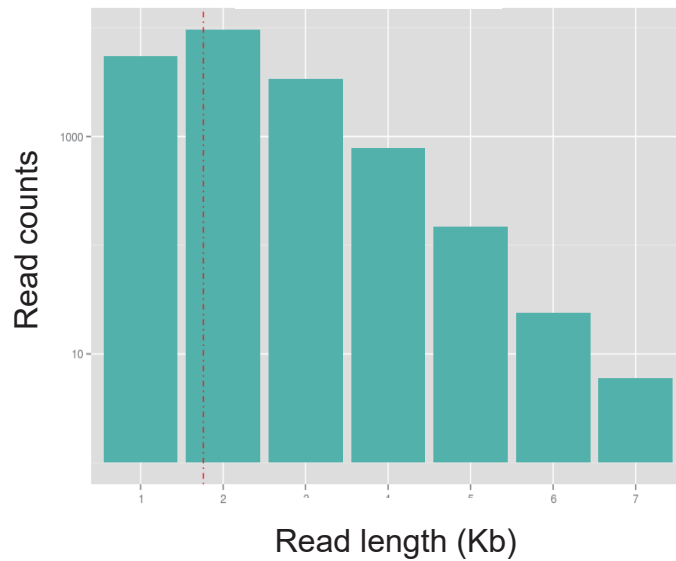

Supplement: Supplementary Figure 1 — Quality control and information of full-length reads using GLN011 as an example. (A) Length distribution of clean reads. (B) Length distribution of full-length (FL) reads. (C) Length distribution of consensus reads. (D) Length distribution of non-redundant FL reads. [file Image_1.pdf]

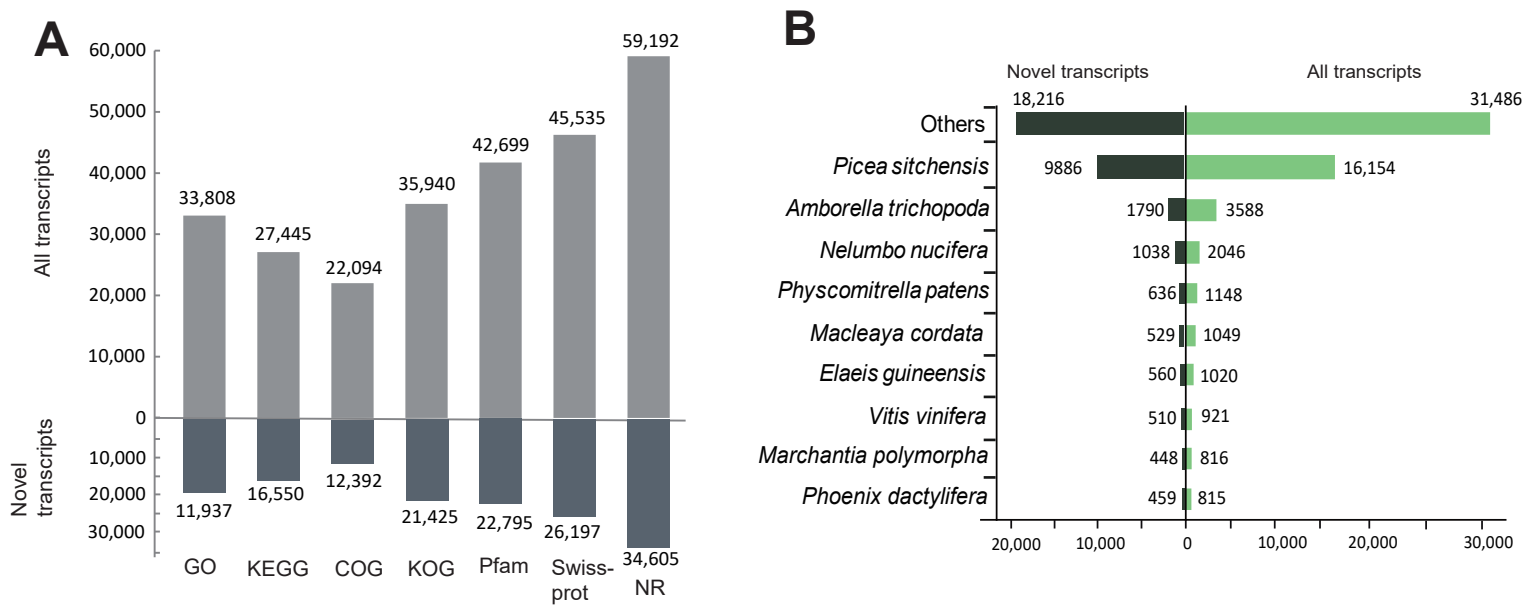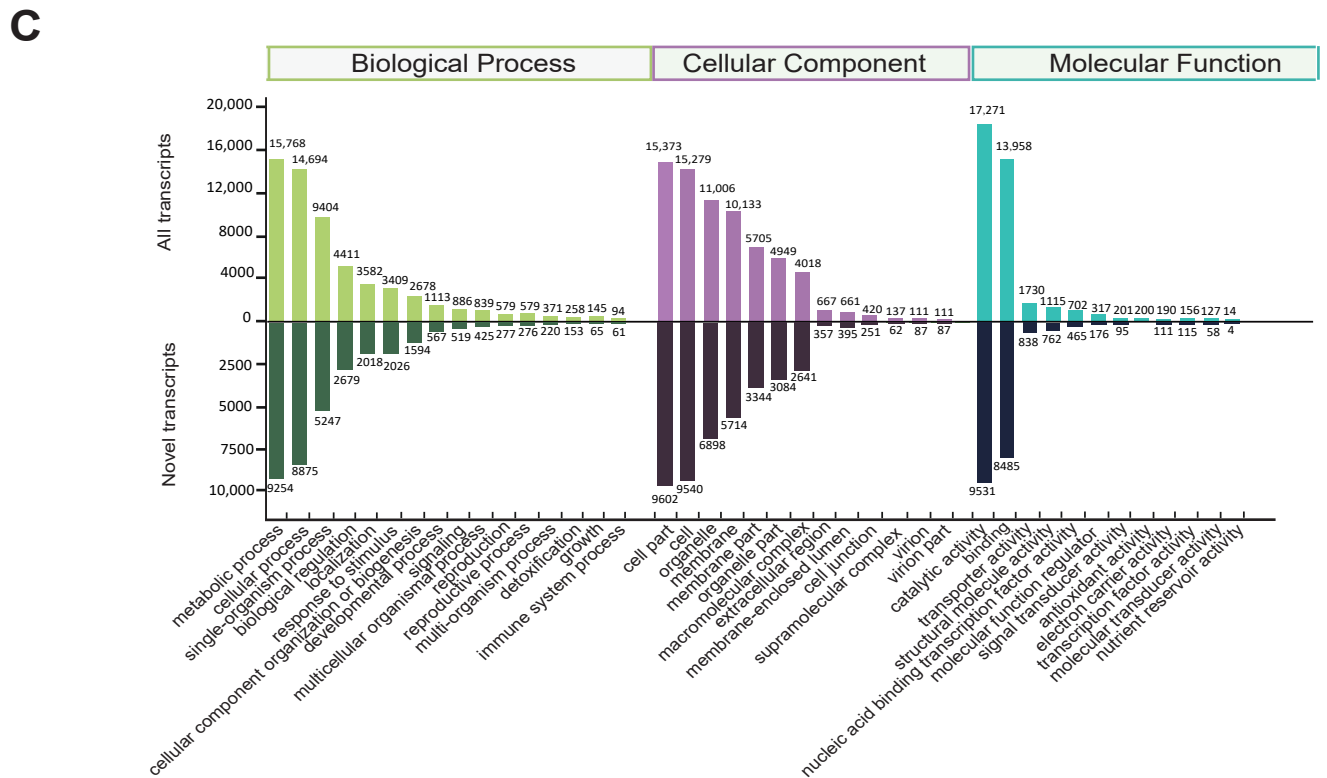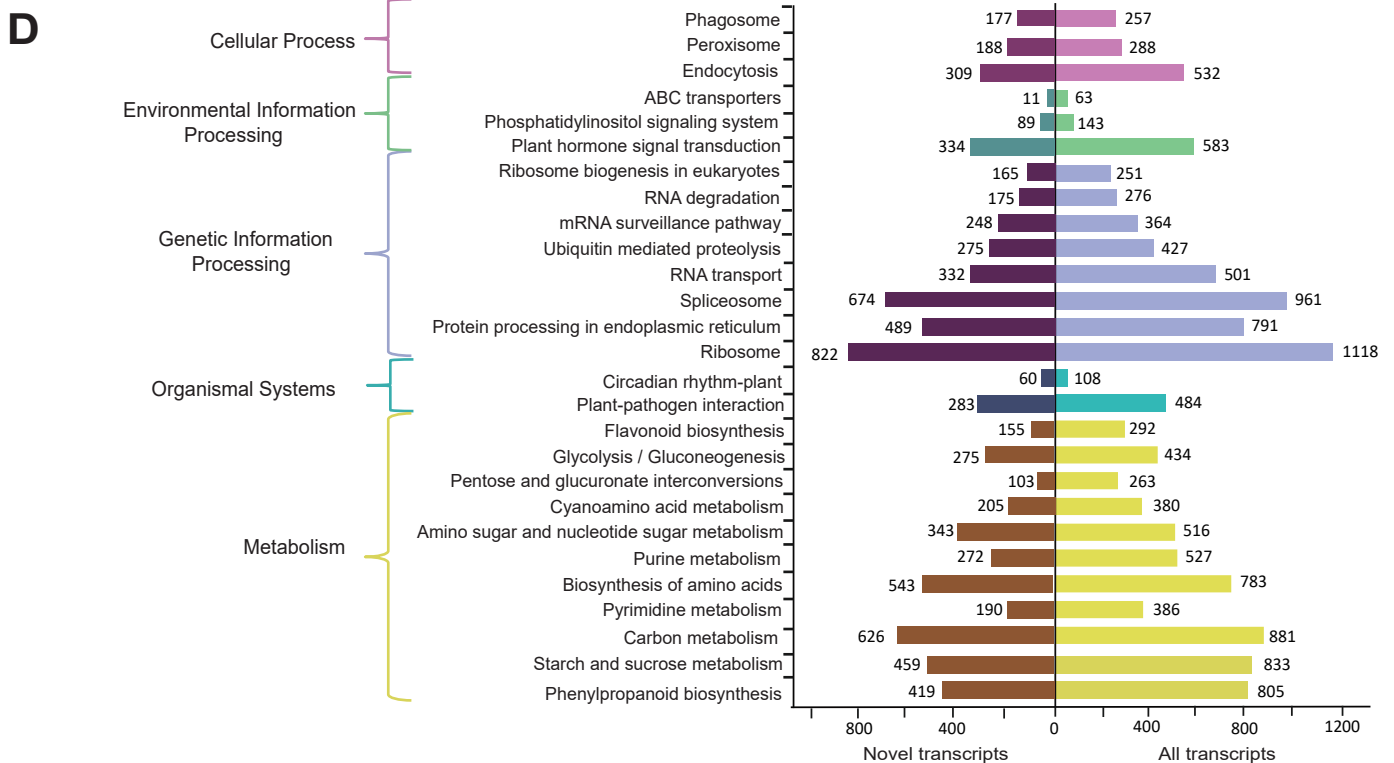

Supplement: Supplementary Figure 2 — All gene and novel gene annotation on the basis of seven databases. (A) Gene number annotated by the seven databases. (B) NR homologous species distribution diagram of all genes and novel genes. (C) Distribution of GO terms for all annotated genes and novel genes. (D) KEGG pathways of all genes and novel genes annotated in the full-length transcriptome of G. luofuense. [file Image_2.pdf]
