# Supplementary material for: Comparative Analyses of Full-Length Transcriptomes Reveal Gnetum luofuense Stem Developmental Dynamics
Source: Front Genet. 2021 Mar 25;12:615284. doi: 10.3389/fgene.2021.615284 (PMC8027257; doi:10.3389/fgene.2021.615284)
Supplement: Supplementary Table 1 — Primer information and qRT-PCR systems. [file Table_1.docx]

**Supplementary Table S1.** Primer information and qRT–PCR systems

| ID | Sequence(5’- 3’) | Product Length(bp) |
| --- | --- | --- |
| G-actin forward sequence (F) | TTGTAGGTCGCCCTCGTC |  |
| G-actin reverse sequence (R) | CTCCCTGTTAGCCTTTGG |  |
| TnS000507375t33-F | CTGCAGAGACTGAGATTGGG | 147 |
| TnS000507375t33-R | CCCCTTGCTTAGCAGAATCC |  |
| TnS000170537t04-F | TCAAAGGGGCTCATCAGACT | 94 |
| TnS000170537t04-R | GCTGGCCTCATCAGATTTCA |  |
| TnS000176189t04-F | CTCCTCGTCTGCAAGTAACC | 207 |
| TnS000176189t04-R | CCTCCGATCTGGGGATTTTG |  |
| TnS000702569t06-F | TTGGGGAATCTGTGAATCAG | 204 |
| TnS000702569t06-R | GGCTCACCTATAGTGTTCTG |  |
| TnS000173151t07-F | GGTCTGAATAGATGCGGCAA | 147 |
| TnS000173151t07-R | TGCTATCTCACACCACCTGT |  |
| TnS000146041t04-F | CATCTGCACAGGATCAGGAC | 169 |
| TnS000146041t04-R | AGCTCTCGGAGTTTTTCGTC |  |
| TnS000991505t06-F | AGGAGGATCTCATCTCTAGG | 133 |
| TnS000991505t06-R | CCGCTACTACTAGTCCCAT |  |
| TnS000448505t01-F | GGGTGAATCAACGTCGTGTA | 250 |
| TnS000448505t01-R | TGCTGTGCTAGTATTGGCTG |  |
| TnS000798047t02-F | TAAGCCCTTTGAAGGCCAAG | 186 |
| TnS000798047t02-R | CACCCGAGGTACAATCAAGG |  |

| Bestar® SybrGreen qPCRmasterMix | 10 μl |
| --- | --- |
| PCR Forward Primer（10μM） | 0.5μl |
| PCR Reverse Primer（10μM） | 0.5μl |
| cDNA template | 1μl |
| ddH_2_O | 8μl |
| Bestar® SybrGreen qPCRmasterMix | 5μl |
