# Supplementary material for: Comparative Analyses of Full-Length Transcriptomes Reveal Gnetum luofuense Stem Developmental Dynamics
Source: Front Genet. 2021 Mar 25;12:615284. doi: 10.3389/fgene.2021.615284 (PMC8027257; doi:10.3389/fgene.2021.615284)
Supplement: Supplementary Table 2 — Clean reads generated by Nanopore sequencing. [file Table_2.docx]

**Supplementary Table S2.** Clean reads generated by Nanopore sequencing

| Sample name | Number of raw reads | Number of base pairs (bp) | N50 | Mean length  (bp) | Maximum  length (bp) |
| --- | --- | --- | --- | --- | --- |
| GLN011 | 3,344,598 | 4,429,311,613 | 1,485 | 1,324 | 12,140 |
| GLN012 | 3,574,191 | 4,809,201,543 | 1,526 | 1,345 | 12,418 |
| GLN013 | 3,761,576 | 5,034,440,488 | 1,481 | 1,338 | 12,021 |
| GLN021 | 4,713,628 | 5,829,710,664 | 1,377 | 1,236 | 17,129 |
| GLN022 | 3,551,523 | 4,573,854,970 | 1,449 | 1,287 | 12,854 |
| GLN023 | 3,436,453 | 4,156,101,125 | 1,323 | 1,209 | 11,183 |
| GLN031 | 5,178,827 | 6,467,288,269 | 1,393 | 1,248 | 11,944 |
| GLN032 | 3,691,362 | 4,795,153,891 | 1,486 | 1,299 | 12,594 |
| GLN033 | 4,792,269 | 6,061,312,556 | 1,416 | 1,264 | 12,167 |
| GLN041 | 3,607,556 | 4,515,036,058 | 1,407 | 1,251 | 13,322 |
| GLN042 | 4,454,381 | 5,708,040,955 | 1,456 | 1,281 | 13,079 |
| GLN043 | 4,050,087 | 5,238,145,047 | 1,466 | 1,293 | 16,315 |
