# Supplementary material for: Comparative Analyses of Full-Length Transcriptomes Reveal Gnetum luofuense Stem Developmental Dynamics
Source: Front Genet. 2021 Mar 25;12:615284. doi: 10.3389/fgene.2021.615284 (PMC8027257; doi:10.3389/fgene.2021.615284)
Supplement: Supplementary Table 3 — Numbers and percentages of FL reads. [file Table_3.docx]

**Supplementary Table S3.** Numbers and percentages of FL reads

| Sample name | Number of clean reads (except rRNA) | Number of full-length reads | Full-length percentage (FL%) |
| --- | --- | --- | --- |
| GLN011 | 3,328,135 | 2,541,173 | 76.35% |
| GLN012 | 3,556,555 | 2,862,650 | 80.49% |
| GLN013 | 3,743,332 | 3,043,575 | 81.31% |
| GLN021 | 4,693,025 | 3,839,899 | 81.82% |
| GLN022 | 3,534,435 | 2,883,701 | 81.59% |
| GLN023 | 3,419,059 | 2,736,218 | 80.03% |
| GLN031 | 5,155,064 | 4,165,530 | 80.80% |
| GLN032 | 3,674,337 | 2,952,748 | 80.36% |
| GLN033 | 4,768,781 | 3,897,533 | 81.73% |
| GLN041 | 3,591,444 | 2,887,529 | 80.40% |
| GLN042 | 4,433,876 | 3,590,727 | 80.98% |
| GLN043 | 4,030,449 | 3,233,961 | 80.24% |
