# Supplementary material for: Comparative Analyses of Full-Length Transcriptomes Reveal Gnetum luofuense Stem Developmental Dynamics
Source: Front Genet. 2021 Mar 25;12:615284. doi: 10.3389/fgene.2021.615284 (PMC8027257; doi:10.3389/fgene.2021.615284)
Supplement: Supplementary Table 4 — Mapped FL reads. [file Table_4.docx]

**Supplementary Table S4.** Mapped FL reads

| Sample | Total Reads | Mapped Reads | Mapped Rates % |
| --- | --- | --- | --- |
| GLN011 | 3,328,135 | 3,317,956 | 99.69% |
| GLN012 | 3,556,555 | 3,546,383 | 99.71% |
| GLN013 | 3,743,332 | 3,733,604 | 99.74% |
| GLN021 | 4,693,025 | 4,671,115 | 99.53% |
| GLN022 | 3,534,435 | 3,521,421 | 99.63% |
| GLN023 | 3,419,059 | 3,399,666 | 99.43% |
| GLN031 | 5,155,064 | 5,131,246 | 99.54% |
| GLN032 | 3,674,337 | 3,658,616 | 99.57% |
| GLN033 | 4,768,781 | 4,755,113 | 99.71% |
| GLN041 | 3,591,444 | 3,576,158 | 99.57% |
| GLN042 | 4,433,876 | 4,413,434 | 99.54% |
| GLN043 | 4,030,449 | 4,014,681 | 99.61% |
