# Supplementary material for: Comparative Analyses of Full-Length Transcriptomes Reveal Gnetum luofuense Stem Developmental Dynamics
Source: Front Genet. 2021 Mar 25;12:615284. doi: 10.3389/fgene.2021.615284 (PMC8027257; doi:10.3389/fgene.2021.615284)
Supplement: Supplementary Table 5 — Information of consensus reads. [file Table_5.docx]

**Supplementary Table S5.** Information of consensus reads

| Sample name | Number of consensus reads | Bps | N50 | Mean length  (bp) | Maximum length (bp) |
| --- | --- | --- | --- | --- | --- |
| GLN011 | 34843 | 50,226,700 | 1,683 | 1,441 | 6,601 |
| GLN012 | 37383 | 54,392,450 | 1,696 | 1,455 | 6,181 |
| GLN013 | 37473 | 55,712,842 | 1,735 | 1,486 | 6,838 |
| GLN021 | 47476 | 63,442,871 | 1,592 | 1,336 | 6,351 |
| GLN022 | 37999 | 53,251,736 | 1,649 | 1,401 | 7,328 |
| GLN023 | 37080 | 47,538,347 | 1,499 | 1,282 | 6,062 |
| GLN031 | 49929 | 67,733,979 | 1,618 | 1,356 | 6,581 |
| GLN032 | 41974 | 57,608,682 | 1,637 | 1,372 | 6,668 |
| GLN033 | 42869 | 59,779,832 | 1,644 | 1,394 | 7,485 |
| GLN041 | 39637 | 52,525,807 | 1,574 | 1,325 | 7,800 |
| GLN042 | 45337 | 63,759,056 | 1,677 | 1,406 | 7,718 |
| GLN043 | 42859 | 58,920,953 | 1,635 | 1,374 | 7,312 |
