# Supplementary material for: Comparative Analyses of Full-Length Transcriptomes Reveal Gnetum luofuense Stem Developmental Dynamics
Source: Front Genet. 2021 Mar 25;12:615284. doi: 10.3389/fgene.2021.615284 (PMC8027257; doi:10.3389/fgene.2021.615284)
Supplement: Supplementary Table 6 — Statistics of non-redundant consensus transcripts. [file Table_6.docx]

**Supplementary Table S6.** Statistics of non-redundant consensus transcripts

| Sample name | Number of non-redundant  transcripts | Number of base pairs (bp) | N50 | Mean length  (bp) | Maximum length (bp) |
| --- | --- | --- | --- | --- | --- |
| GLN011 | 19,360 | 29,392,453 | 1762 | 1518 | 6601 |
| GLN012 | 20,385 | 31,120,972 | 1769 | 1526 | 6113 |
| GLN013 | 20,797 | 32,271,512 | 1801 | 1551 | 6838 |
| GLN021 | 24,349 | 35,383,552 | 1706 | 1453 | 6351 |
| GLN022 | 20,453 | 30,705,067 | 1746 | 1501 | 7328 |
| GLN023 | 19,528 | 27,318,061 | 1616 | 1398 | 6062 |
| GLN031 | 25,608 | 37,699,117 | 1726 | 1472 | 6581 |
| GLN032 | 21,627 | 32,511,064 | 1758 | 1503 | 6668 |
| GLN033 | 22,902 | 34,045,642 | 1739 | 1486 | 7485 |
| GLN041 | 20,739 | 30,152,478 | 1695 | 1453 | 6105 |
| GLN042 | 23,538 | 36,023,706 | 1791 | 1530 | 7718 |
| GLN043 | 22,020 | 33,201,405 | 1767 | 1507 | 7312 |
